# Osteogenesis and Morphology of the Peri-Implant Bone Facing Dental Implants

**DOI:** 10.1100/tsw.2004.211

**Published:** 2004-12-14

**Authors:** Marco Franchi, Ester Orsini, Alessandra Triré, Marilisa Quaranta, Desirée Martini, Gabriella Giuliani Piccari, Alessandro Ruggeri, Vittoria Ottani

**Affiliations:** Dipartimento di Scienze Anatomiche Umane e Fisiopatologia dell'Apparato Locomotore, Universitài Bologna, Via Irnerio 48, 40126 Bologna, Italy

**Keywords:** implant surface, peri-implant osteogenesis, early implant healing, histology, ultrastructure

## Abstract

This study investigated the influence of different implant surfaces on peri-implant osteogenesis and implant face morphology of peri-implant tissues during the early (2 weeks) and complete healing period (3 months). Thirty endosseous titanium implants (conic screws) with differently treated surfaces (smooth titanium = SS, titanium plasma sprayed = TPS, sand-blasted zirconium oxide = Zr-SLA) were implanted in femur and tibiae diaphyses of two mongrel sheep. Histological sections of the implants and surrounding tissues obtained by sawing and grinding techniques were observed under light microscopy (LM). The peri-implant tissues of other samples were mechanically detached from the corresponding implants to be processed for SEM observation. Two weeks after implantation, we observed osteogenesis (new bone trabeculae) around all implant surfaces only where a gap was present at the host bone-metal interface. No evident bone deposition was detectable where threads of the screws were in direct contact with the compact host bone. Distance osteogenesis predominated in SS implants, while around rough surfaces (TPS and Zr-SLA), both distance and contact osteogenesis were present. At SEM analysis 2 weeks after implantation, the implant face of SS peri-implant tissue showed few, thin, newly formed, bone trabeculae immersed in large, loose, marrow tissue with blood vessels. Around the TPS screws, the implant face of the peri-implant tissue was rather irregular because of the rougher metal surface. Zr-SLA screws showed more numerous, newly formed bone trabeculae crossing marrow spaces and also needle-like crystals in bone nodules indicating an active mineralising process. After 3 months, all the screws appeared osseointegrated, being almost completely covered by a compact, mature, newly formed bone. However, some marrow spaces rich in blood vessels and undifferentiated cells were in contact with the metal surface. By SEM analysis, the implant face of the peri-implant tissue showed different results. Around the SS screws, the compact bone with areas of different mineralisation rate appeared very smooth, while around the rougher TPS screws, the bone still showed an irregular surface corresponding to the implant macro/microroughness. Around the Zr-SLA screws, a more regular implant-bone surface and sparse, calcified marrow spaces were detectable.Results from this research suggest that 2 weeks after implantation, trabecular bone represents the calcified healing tissue, which supports the early biological fixation of the implants. The peri-implant marrow spaces, rich in undifferentiated cells and blood vasculature, observed both 2 weeks and 3 months after surgery, favour the biological turnover of both early and mature peri-implant bone. The implant surface morphology strongly influences the rate and the modality of peri-implant osteogenesis, as do the morphology and arrangement of the implant face in peri-implant bone both during early healing (after 2 weeks) and when the implant is just osseointegrated; rough surfaces, and in particular Zr-SLA, seem to better favour bone deposition on the metal surface.

## INTRODUCTION

The insertion of endosseous dental implants in jaws with a direct bone-to-metal contact is a widely accepted treatment modality for prosthodontic rehabilitation of fully or partially edentulous patients[1]. A direct bone-to-implant contact is defined as “osseointegration” and consists of a functional ankylosis of the device in the newly formed bone. Implant osseointegration is the final goal of implant surgery and the prerequisite to achieve long-term success of endosseous dental implants[2,3].

When an implant is placed in a bone cavity, the healing process develops on the way to final implant osseointegration. Histological events in the bone formation process up to osseointegration have been widely investigated[4,5,6,7,8,9]. Immediately after the insertion of titanium implants in rat maxilla and dog mandible, the bone-implant interface is rapidly filled by a blood clot, red cells, inflammatory cells mainly consisting of neutrophils, and degenerating cellular elements[6,7]. In 3–5 days after implant placement, a layer of flattened cells with interposed blood capillaries lines the implant surface, the intercellular spaces being filled with fibrin. In 5–7 days after implantation, cuboidal cells cover the implant surface and the osteoid/new bone tissue formation develops around the implant surface[10], but it has been reported that just 3 days after implantation some small needle-like crystals are detectable directly at the implant surface[9]. The new bone deposition proceeds until 28 days after surgery; the implant appears well osseointegrated[10]. The establishment of osseointegration has been described through a new peri-implant lamellar bone[6], or a primary spongiosa rich in vascular structures[7], or a trabecular bone with wide marrow spaces[11]. The implant surface strongly influences the modality of peri-implant bone osteogenesis; rough surfaces favour new bone deposition directly on the implant surface (direct osteogenesis or *de novo* bone formation) and not only from the host bone toward the implant (distance osteogenesis)[1,7,8,9,12]. Moreover, new bone formation has been induced in a nonosseous site, such as dorsal muscles of beagle dogs, by porous titanium that was believed to be a nonbioactive material[13]. So it has been suggested that implant healing probably depends on surface properties and quality of the implant[10,14].

The aim of our investigation was to establish the influence of differently treated implant surfaces on osteogenesis and morphology of peri-implant tissues at the implant interface during the early (2 weeks) and complete (3 months) healing period.

## MATERIALS AND METHODS

Thirty endosseous titanium implant conic screws (3.8 mm outer diameter and 8 mm long) (Or-Vit Castelmaggiore-Bologna, Italy) with different surfaces (smooth titanium = SS, titanium plasma sprayed = TPS, sand-blasted zirconium oxide = Zr-SLA) were implanted in femur and tibiae diaphyses of two mongrel sheep (3–4 years old) at two different times.

Animals were anaesthetised according to a standardised protocol: premedication with intramuscular injection of 10 mg/kg body weight ketamine (Ketavet 100, Farmaceutici Gellini, SpA, Aprilia, Italy), 0.3 mg/kg body weight xylazine (Rompun, Bayer AG, Leverkusen, Germany), and subcutaneous injection of 0.0125 mg/kg body weight atropine sulphate; induction with intravenous injection of 6 mg/kg body weight sodium thiopentone (2.5% solution, Pentothal, Hoechst AG, Germany); maintenance with $O_2$, $N_2O$, and 1–2.5% halothane under assisted ventilation (Servo Ventilator 900 D, Siemens, Germany). A 3.5-mm-diameter drill was used to predrill the holes in each diaphysis.

Antibiotics (cephalosporin, 1 g/day for 5 days) and analgesics (ketoprofen 500 mg/day for 3 days) were administered postoperatively.

The sheep were sacrificed with intravenous administration of Tanax (Hoechst, Frankfurt am Main, Germany) under general anaesthesia when 15 implants were 14 days old and the other 15 were 90 days old. All implants with the surrounding peri-implant tissues were isolated, removed, and fixed in 10% formalin-buffered solution (pH 7.2) for histological and ultrastrucural analyses. Eighteen samples (9 screws dated 15 days after implantation; 9 implants 90 days after insertion) were then dehydrated in ethanol and embedded in methyl methacrylate. There were 60- to 100-μm-thick sections obtained by sawing and grinding techniques (Saw and Grinding, Remet, Bologna, Italy), stained with toluidine blue and acid fuchsin, and finally observed under a light microscope.

The other 12 samples (6 aged 15 days and 6 aged 90 days) were processed for SEM observation. After a 30-min fixation with Karnovsky solution (paraformaldehyde 4%, glutaraldehyde 2.5%, 0.1*M* cacodylate buffer), the peri-implant tissues were mechanically detached from the corresponding implants and dehydrated with ethanol, to be finally dried with hexamethyldisilazane (Sigma). The samples corresponding to the peri-implant tissues were mounted on stubs with carbon bioadhesive film, gold/palladium-coated, and observed with a Philips 515 scanning electron microscope (SEM: Philips 515, Eindhoven, Holland) fitted with secondary electron (SE) and back-scattered electron (BSE) probes, at voltages of 15 kV. All the procedures involving the sheep were performed strictly following Italian and European Law on animal experimentation (Decree Law, 27th January; 1992 no. 116-in accordance with the EEC rules and Animal Welfare Assurance No. #A5424-01 of the National Institute of Health [NIH-Rockville, Maryland, USA]).

## RESULTS

No surgical complication was encountered during implant insertion in both sheep and the animals survived the whole postsurgical period without developing any infection.

### Light Microscopy (2 Weeks)

Histological observation of the peri-implant bone around SS screws showed no newly formed bone or connective tissue around threads in direct contact with the host bone. By contrast, the areas interposed between two adjacent threads exhibited a gap (270–450 μm) at the host bone-implant interface. In those areas, thin bone trabeculae developing from the host bone ran towards the metal surface and crossed large, loose, marrow spaces rich in undifferentiated cells and a wide network of blood vessels. Sometimes, small host bone chips, occasionally arisen from the action bur during cavity preparation, were partially enclosed by the bone trabeculae (Fig. 1).

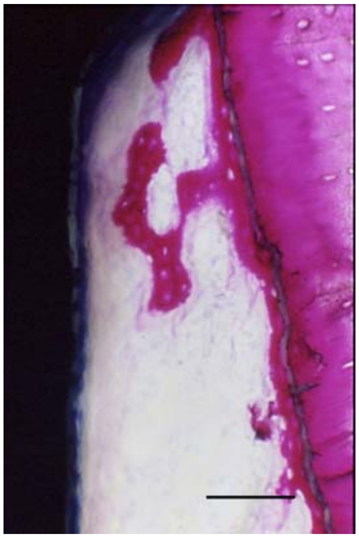

**FIGURE 1:** LM image of SS screw 2 weeks after surgery. Thin bone trabeculae developing from the host bone run towards the metal surface and cross large loose marrow spaces rich in undifferentiated cells and a wide network of blood vessels. Bar = 100 µm.

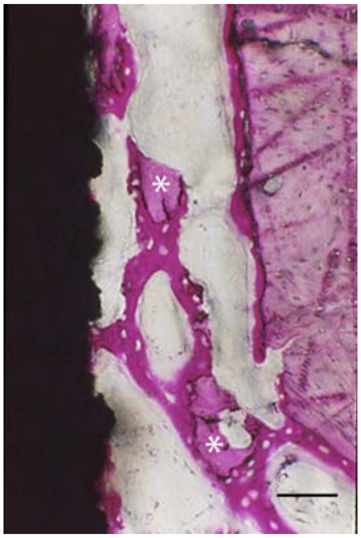

**FIGURE 2.** LM image of TPS screw 2 weeks after surgery. Newly formed bone trabeculae and osteoid tissue with osteoblasts are visible both rising from the host bone and holding on to the rough implant surface. Small host bone chips (*), occasionally arisen from the action bur during cavity preparation, are partially enclosed in the new bone trabeculae. Bar = 100 µm.

Peri-implant tissue around TPS screws showed similar results, but the newly formed bone trabeculae were visible both rising from the host bone and holding onto the rough implant surface. A thick layer of osteoid tissue and many active osteoblasts covered the surface of the bone trabeculae. A line of flattened-rounded cells appeared to hold onto some areas of the implant surface. Many blood vessels were visible next to the implant surface (Fig. 2).

A gap similar to that observed in the other samples was present in areas between two adjacent threads of Zr-SLA screws. In those regions, a network of many newly formed bone trabeculae developed both from host bone and directly on the metal surface. These thin bone trabeculae often ran parallel and on the

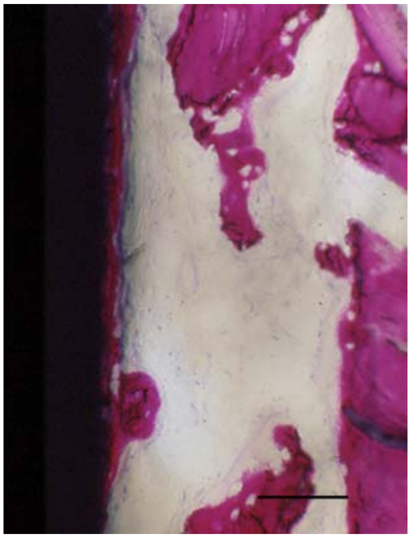

**FIGURE 3**. LM image of Zr-SLA screw 2 weeks after surgery. Newly formed bone trabeculae develop both from host bone (on the right) and directly on the metal surface. Many osteoblasts are lined on these bone trabeculae, which contain rounded osteoblasts/osteocytes in wide lacunae. Bar = 100 µm.

metal surface; they were rich in rounded osteoblasts/osteocytes in wide lacunae and sometimes included host bone chips (35–220 µm) (Fig. 3).

## SE and BSE Analysis (2 Weeks)

Scanning electron analysis of implant face of peri-implant bone around SS screws 2 weeks after placement confirmed the histological data. In areas between two adjacent threads, a few newly formed, thin bone trabeculae with wide cellular lacunae developed from host bone towards the implant surface. Many blood vessels in loose marrow spaces ran next to the titanium surface. Osteoblasts/osteocytes-like cells were held on an osteoid collagen fibre network and appeared in some cases partially included by the calcifying front. A compact old bone with osteocytes in bone lacunae or marrow spaces with blood vessels were observed in proximity of the implant threads (Fig. 4).

Peri-implant hard tissue around TPS screws in areas between two threads exhibited a very irregular surface with short bone trabeculae, often orthogonally arranged to the metal surface and interposed to marrow spaces (Fig. 5). At the thread interface, old compact bone was often observed.

The implant face of Zr-SLA peri-implant bone between two threads showed newly formed bone trabeculae rich in osteoblasts/osteocytes in their lacunae and marrow spaces with many blood vessels. Needle-like crystals arranged in bone-like nodules were occasionally observed on the implant face of bone next to the removed implant surface (Fig. 6).

## Light Microscopy (3 Months)

Three months after implantation, the peri-implant tissues surrounding SS, TPS, and Zr-SLA screws showed similar histological aspects in all samples. In fact, the peri-implant bone in direct contact with the implant surface was represented by a mature lamellar bone with evident osteons. This bone covered most of the implant surface that appeared well osseointegrated, but small marrow spaces with a rich vasculature were sometimes in direct contact with the metal. In particular, in these regions, a line of rounded cells appeared directly held on the titanium surface (Figs. 7, 8, and 9).

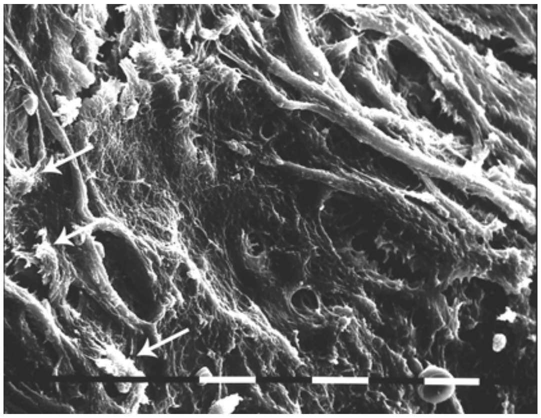

**FIGURE 4.** SEM micrograph of peri-implant tissue around SS implant 2 weeks after surgery. An osteoid tissue with calcifying collagen fibres observed around the implant surface between 2 threads. Osteocytes and rounded osteoblast-like cells appear to hold on to the collagen fibre network and partially included by the calcifying front (arrows). Bar = 10 µm.

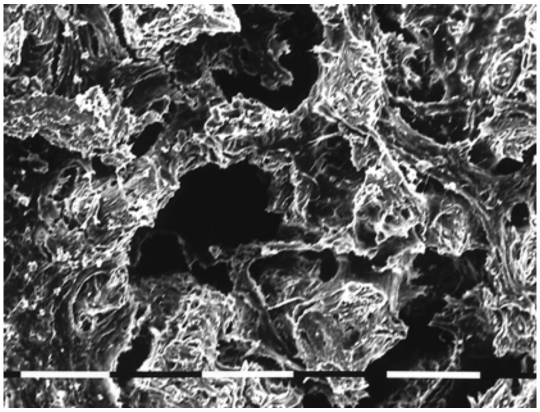

**FIGURE 5**. SEM micrograph of peri-implant tissue around TPS implant 2 weeks after surgery. A very irregular surface of short bone trabeculae orthogonally arranged to the metal surface with cellular lacunae and small marrow spaces is evident in direct contact with the implant surface between 2 threads. Bar = 10 µm.

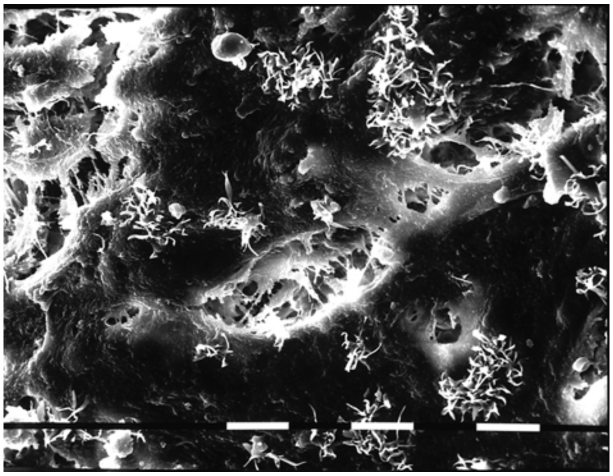

**FIGURE 6.** SEM micrograph of peri-implant tissue around Zr-SLA implant 2 weeks after surgery. Newly formed bone with osteoblasts in bone lacunae and needle-like crystals arranged in bone-like nodules next to the removed implant surface. Bar = 10 µm.

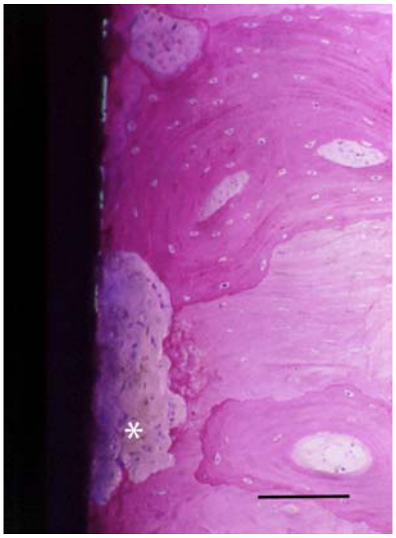

**FIGURE 7**. LM image of SS screw 3 months after surgery. The peri-implant bone in direct contact with the implant is a mature lamellar bone with osteons. A small marrow space (*) rich in blood vessels and cells is visible in direct contact with the metal. Bar = 100 µm.

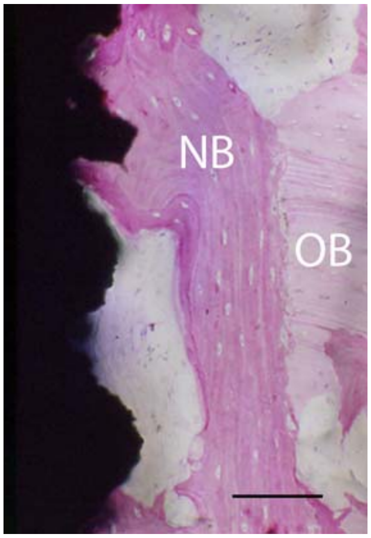

**FIGURE 8**. LM image of TPS screw 3 months after surgery. Newer peri-implant bone (NB) is in direct contact with the implant surface. A marrow space with a rich vasculature appears in direct contact with the metal. Older bone (OB). Bar = 100 µm.

## SE and BSE Analysis (3 Months)

The ultrastrucutural analysis of the implant face of peri-implant tissue showed different morphological aspects around SS, TPS, and Zr-SLA screws. In particular, the compact mature bone around the SS implants between two threads appeared very smooth, with few ridges congruent to the grooves of machining treatment. Areas of bone with different density corresponded to a different rate/age of mineralisation (Fig. 10 a, b). In some areas, a trabecular bone with small marrow spaces developed from an underlying compact bone.

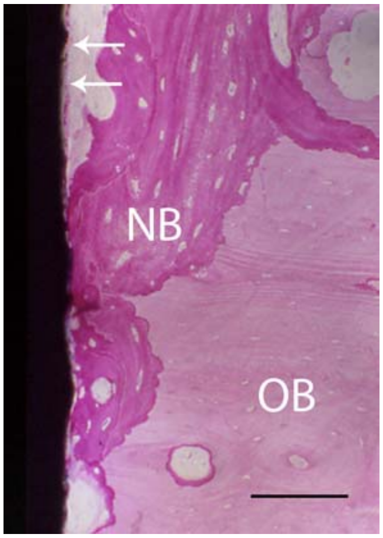

**FIGURE 9**. LM image of Zr-SLA screw 3 months after surgery. Newly formed peri-implant mature lamellar bone in direct contact with the implant surface (NB). Older bone (OB). Small marrow spaces with blood vessels are in direct contact with the metal. In these regions a line of rounded cells appear to intimately hold on the titanium surface (arrows). Bar = 100 µm
.

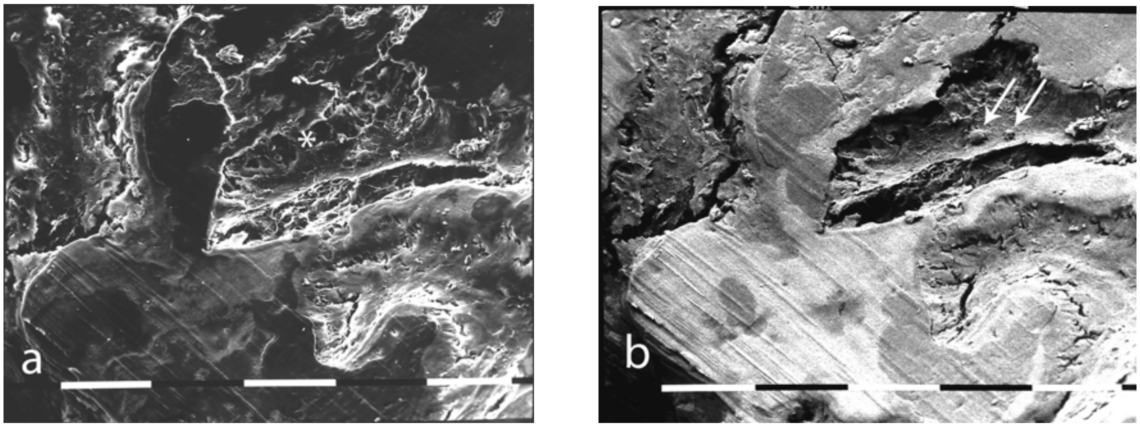

**FIGURE 10.** SE (a) and BSE (b) images of peri-implant tissue around SS implant 3 months after surgery. Newly formed compact bone can be seen in direct contact with the removed implant surface. Straight calcified ridges (down on the left) correspond to the implant machining treatment. Different density areas of mature bone correspond to different calcification ages. Marrow spaces (*) with cells (arrows) and blood vessels are detectable. Bar = 10 µm.

The implant face of peri-implant compact bone around TPS screws was irregular because of the presence of small bone concavities complementary to the irregular TPS implant surface (Fig. 11). Microscopic holes corresponding to microroughness of the removed implant surface were recurrent (Fig. 12).

SEM analysis between two adjacent threads of Zr-SLA screws demonstrated a peri-implant lamellar bone in direct contact with the removed implant surface. A network of small, thin, residual marrow spaces containing fibrous tissue were better discernable by BSE analysis (Fig. 13 a, b). Sometimes, residual marrow spaces appeared filled with a calcified tissue.

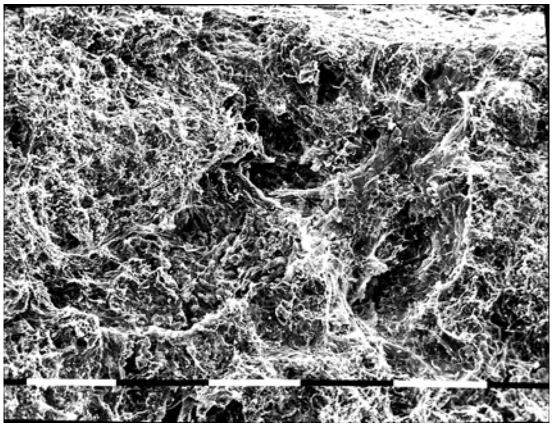

**FIGURE 11**. SEM micrograph of peri-implant tissue in direct contact with the removed TPS screw 3 months after surgery. A rough surface of mineralized mature bone with a small bone concavity complementary to the irregular TPS surface can be seen in the areas between 2 threads. Bar = 10 µm.

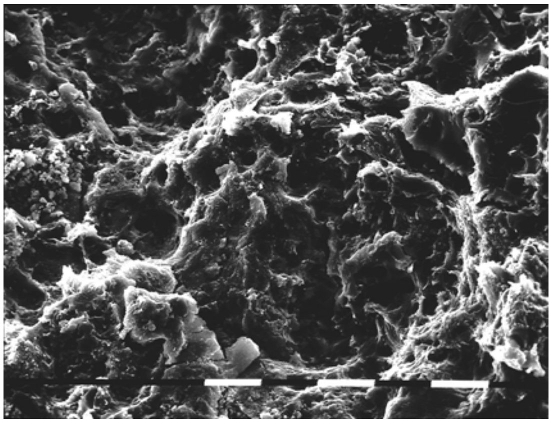

**FIGURE 12**. SEM micrograph of peri-implant tissue around TPS implant 3 months after surgery. A bone concavity of mature lamellar peri-implant bone shows microscopic holes corresponding to the microroughness of the removed implant surface. Bar = 10 µm.

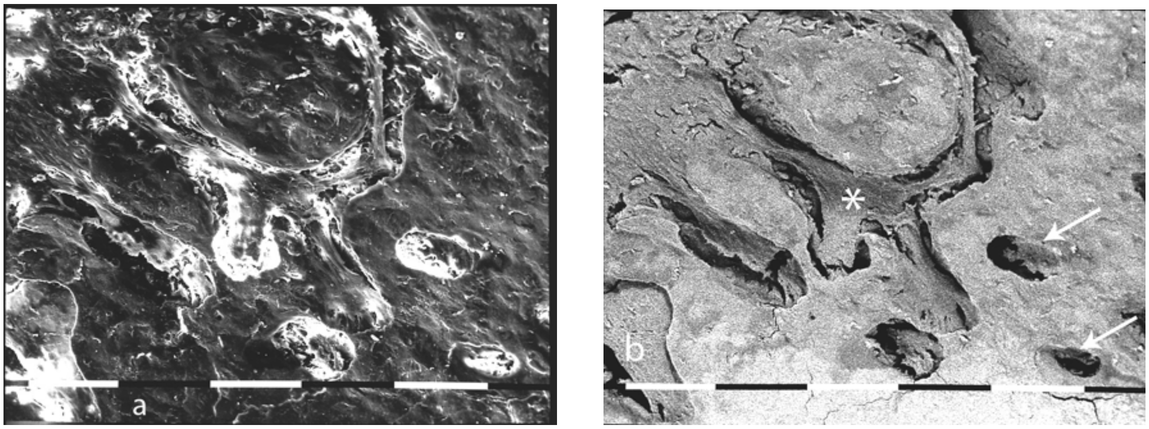

**FIGURE 13**. SE (a) and BSE (b) images of peri-implant tissue around Zr-SLA implant 3 months after surgery. Some areas of mature lamellar bone in direct contact with the implant surface and thin residual marrow spaces (*) with cell lacunae (arrows) are detectable. Bar = 10 µm.

## DISCUSSION

Although most implant clinical failures occur at the early stages of peri-implant healing[15,16], most histological studies of implants investigated the bone-implant interface after a relatively long period; i.e., when the implants appeared just osseointegrated with a mature bone to allow functional loading[1,17,18].

Dental implants are now increasingly loaded immediately or after a short period of healing to improve patient morbidity and decrease healthcare costs[9,19,20,21,22,23,24,25].

Immediately after the insertion of an endosseous implant, a gap between the metal surface and host bone could be appropriate for a rapid deposition of new bone[6,7,10,11], even if large gaps (more than 0.5 mm) can reduce the quality of new peri-implant bone and delay the rate of filling[17]. According to this research, this study disclosed osteogenesis 2 weeks after implantation around all the implant surfaces tested (SS, TPS, and Zr-SLA) only where a gap was present at the host bone-metal interface. No evident bone deposition was detectable where the threads of the screws were in direct contact with the compact host bone. In particular, histological results suggested that a trabecular bone was still present 14 days after surgery around all implants, but while in SS implants a distance osteogensis predominated, around rough surfaces (TPS and Zr-SLA), both distance and contact osteogenesis were present[1,7,9,12,26,27]. The aim of peri-implant early healing is to fill the gap between the host bone and the implant rapidly and with functional calcified tissue. The healing trabecular bone is the proper intermediate peri-implant healing bone as it fills the peri-implant space far more quickly[8] and represents a valid biological anchorage of the implant in the bone cavity. This tissue will be then substituted by a compact mature bone.

Buser et al.[28] demonstrated that the removal torque values tested on rough-surfaced implants inserted in maxilla of mini pigs were similar at 4, 8, and 12 weeks.

These data suggest that implant fixation is functionally similar in these different periods and that 4 weeks after surgery, the implants mechanically respond as osseointegrated.

At SEM analysis 2 weeks after insertion, the implant face of peri-implant tissue differed in relation to the implant surface morphology. The peri-implant tissue around the SS screws showed a few, thin, newly formed bone trabeculae immersed in loose marrow tissue with blood vessels. Around the TPS screws, the implant face of peri-implant tissue was rather irregular because short new bone trabeculae in contact with the implant surface were interposed by marrow spaces. Zr-SLA screws showed more numerous, newly formed bone trabeculae crossing marrow spaces and needle-like crystals in bone nodules indicating an active mineralising process[9].

After 3 months, all the screws appeared osseointegrated, being almost completely covered by a compact, mature, newly formed bone. Others have reported that after a long period, such as 3 months of healing, peri-implant tissues showed areas of fibrous tissue alternating with direct bone contact[18,29]. In our observations, the described peri-implant connective tissue corresponds to a marrow tissue in marrow spaces. The presence of a highly vascularised tissue rich in undifferentiated cells is helpful for the biological turnover of the peri-implant bone. Marrow spaces have always been observed around and next to the different implant surfaces; they were wide and filled by a loose connective tissue in samples of 14 days, but diminished and appeared reduced in size or as small canals often filled by dense connective tissue or calcified tissue in 3-month-old samples. Their presence next to the implant surface during early peri-implant healing ensures a biological support for the turnover of mineralised tissues; during implant healing bone matrix mineralises and envelops the osteoblasts, which produce the osteoid tissue and, if new bone is required, new osteogenic cells must migrate to that surface[8].

Histological results 3 months after implantation did not disclose major differences among the implant surfaces. On the contrary, SEM analysis of the implant face of the peri-implant tissue showed interesting data. Around the SS screws, the compact bone with areas of different mineralisation rate appeared very smooth, recalling the morphological aspect of the implant surface. Around the very rough TPS screws, the bone still exhibited an irregular surface corresponding to the implant macro/microroughness, while around the Zr-SLA screws, a more regular implant bone surface with calcified marrow spaces was detectable.

In conclusion our findings suggest that:

1. Trabecular bone represents the calcified healing tissue, which could mechanically and biologically support the early biological fixation of implants.
2. Peri-implant marrow spaces, rich in undifferentiated cells and blood vasculature, are still detectable at the implant surface 3 months after implantation and can favour the biological turnover of the peri-implant bone.
3. The implant surface morphology influences the rate and modalities of peri-implant osteogenesis during early healing (after 2 weeks); rough surfaces (in particular Zr-SLA) seem to favour bone deposition on the metal surface.
4. The morphology and arrangement of the implant face in peri-implant bone are related to the morphology of the implant surface also when the implants are just osseointegrated (after 3 months without loading).

## ACKNOWLEGEMENTS

The authors thank OR-Vit (Castelmaggiore, Bologna, Italy) for providing the implants and Dr. Beatrice Bacchelli and Mr. Massimo Gamberini for their technical assistance. This research project was supported by a grant from the University of Bologna, quota ex 60%.
